# Increased cardiovascular risk in patients with chronic kidney disease

**DOI:** 10.1007/s00059-024-05235-4

**Published:** 2024-02-28

**Authors:** Sonja Vondenhoff, Stefan J. Schunk, Heidi Noels

**Affiliations:** 1 Institute for Molecular Cardiovascular Research (IMCAR), RWTH Aachen University, 52074 Aachen, Germany; 2 Biochemistry Department, Cardiovascular Research Institute Maastricht (CARIM), Maastricht University, Maastricht, The Netherlands; 3 Klinik für Innere Medizin IV, Nieren- und Hochdruckkrankheiten, Universitätsklinikum des Saarlandes, Homburg/Saar, Germany; 4 Aachen-Maastricht Institute for Cardiorenal Research (AMICARE), University Hospital Aachen, Aachen, Germany; 5 Institute for Molecular Cardiovascular Research (IMCAR), University Hospital RWTH Aachen University, 52074 Aachen, Germany

**Keywords:** Cardiorenal syndrome, Chronic kidney disease, Cardiovascular diseases, Risk factors, Mechanisms, Kardiorenale Syndrome, Chronische Niereninsuffizienz, Herz-Kreislauf-Erkrankungen, Risikofaktoren, Mechanismen

## Abstract

Cardiovascular disease (CVD) is highly prevalent in patients suffering from chronic kidney disease (CKD). The risk of patients with CKD developing CVD is manifested already in the early stages of CKD development. The impact of declined kidney function on increased cardiovascular risk and the underlying mechanisms are complex and multifactorial. This review discusses the impact of (a) traditional cardiovascular risk factors such as smoking, dyslipidemia, diabetes, and hypertension as well as (b) CKD-specific pathophysiological and molecular mechanisms associated with an increased cardiovascular risk. The latter include uremic toxins, post-translational modifications and uremic lipids, innate immune cell activation and inflammation, oxidative stress, endothelial cell dysfunction, increased coagulation and altered platelet responses, vascular calcification, renin–angiotensin–aldosterone-system (RAAS) and sympathetic activation, as well as anemia. Unraveling the complex interplay of different risk factors, especially in the context of patient subcohorts, will help to find new therapeutic approaches in order to reduce the increased cardiovascular risk in this vulnerable patient cohort.

## Abstract (de)

Herz-Kreislauf-Erkrankungen kommen bei Patienten mit chronischer Niereninsuffizienz sehr häufig vor. Das Risiko von Patienten mit chronischer Niereninsuffizienz, eine kardiovaskuläre Erkrankung zu entwickeln, entsteht bereits in den frühen Stadien der Nierenschädigung. Die Folgen einer verminderten Nierenfunktion für das erhöhte kardiovaskuläre Risiko und die zugrunde liegenden Mechanismen sind komplex und multifaktoriell. Im vorliegenden Review werden zum einen die Auswirkungen herkömmlicher kardiovaskulärer Risikofaktoren wie Rauchen, Dyslipidämie, Diabetes und Bluthochdruck sowie zum anderen für chronische Niereninsuffizienz spezifische pathophysiologische und molekulare Mechanismen erörtert, die mit einem erhöhten kardiovaskulären Risiko in Verbindung gebracht werden. Zu den für chronische Niereninsuffizienz spezifischen Mechanismen gehören urämische Toxine, posttranslationale Modifikationen und urämische Lipide, angeborene Immunzellaktivierung und Entzündung, oxidativer Stress, Dysfunktion der Endothelzellen, erhöhte Gerinnung und veränderte Thrombozytenaktivierung, Gefäßverkalkung, Renin-Angiotensin-Aldosteron-System(RAAS)- und Sympathikusaktivierung sowie Anämie. Die Entschlüsselung dieses komplexen Zusammenspiels verschiedener Risikofaktoren, insbesondere im Kontext von Patientensubkohorten, wird dazu beitragen, neue therapeutische Ansätze zu finden, um das erhöhte kardiovaskuläre Risiko innerhalb dieser gefährdeten Patientenkohorte zu verringern.

## Increased cardiovascular risk in patients with chronic kidney disease

**Fig. 1:**
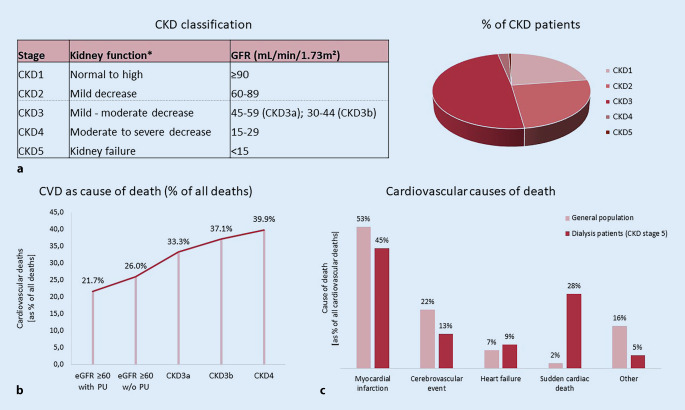
Chronic kidney disease (*CKD*) is highly prevalent in modern society and strongly associated with an increased cardiovascular risk. **a** CKD stages defined by glomerular filtration rate (*GFR*). *Asterisks*: Compared to young adults. Percentage of CKD patients in the respective stages (based on [46]). **b** CVD as cause of death according to CKD stage (adjusted for age and sex; based on [3]). **c** Representation of the most common cardiovascular deaths of CKD5 patients on dialysis compared to the general population. Figures based on [3, 46, 47] and adapted from Baaten et al. [4]. *GFR* glomerular filtration rate, *PU* proteinuria

Around 15–20% of people worldwide suffer from chronic kidney disease (CKD). This greatly increases cardiovascular risk: Whereas 37.5% of individuals with healthy kidney function suffer from cardiovascular disease (CVD), this is increased to 63.4% in CKD patients with only a mild to no reduction in kidney function (CKD stage 1–2); to 66.6% in patients with a mild–moderate or moderate–severe kidney dysfunction (CKD stage 3); and even up to 75.3% in patients with a severely reduced kidney function (CKD stage 4) or complete kidney failure (CKD stage 5; [1]). Cardiovascular mortality increases with declining kidney function (the latter expressed as the estimated glomerular filtration rate, eGFR), also after adjustment for traditional cardiovascular risk factors, indicating CKD as an independent risk factor for CVD [2]. Overall, CVD is the leading cause of death in patients with CKD, with 40–45% of patients with advanced CKD (CKD stage 3b-4) dying from CVD [3] instead of progressing to the end-stage of kidney disease in which patients require kidney transplantation or dialysis (CKD stage 5d; Fig. 1).

## CVD presentation in CKD

As in the general population, myocardial infarction is the leading cause of cardiovascular death in CKD patients, and stroke also remains an important contributor to cardiovascular mortality ([4]; Fig. 1). As underlying pathology, atherosclerosis is more prevalent in CKD and is more likely to progress in patients with progressive CKD [5]. Also, CKD patients show a reduced post-infarction survival [6]. Furthermore, the risk of heart failure, atrial fibrillation, and sudden cardiac death is increased in CKD, with the latter responsible for 28% of cardiovascular deaths in CKD patients compared to only 2% in the general population [1, 4]. Underlying causes include uremic cardiomyopathy characterized by left ventricular hypertrophy and cardiac interstitial fibrosis, as well as cardiac electrical dysregulation triggering arrhythmias [7].

## Traditional risk factors and prevention of CVD in CKD

Traditional cardiovascular risk factors contribute to CVD in CKD and are thus also a focus of prevention and treatment guidelines to reduce cardiovascular risk in CKD patients (Table 1).

**Table 1:** Established therapeutic approaches of the traditional risk factors in CKD [44, 45]

Risk factors of CKD progression	Established therapeutic approaches targeting traditional cardiovascular risk factors in CKD
Blood pressure	Target values <130/80 mm Hg (KDIGO); 130–139 mm Hg syst., 120–129 mm Hg syst. (ESH) If necessary, <120 mm Hg systolic depending on comorbidities and underlying kidney disease RAAS inhibition (maximum tolerated dose) + diuretic and/or calcium channel blocker No combination of angiotensin II receptor blocker and ACE inhibitor in the case of poor risk–benefit profile
Blood sugar	HbA1c target ~ 7.0–8.0% HbA1c target <6.5% in young patients without major comorbidities Metformin and SGLT2-inhibition at first-line therapy after lifestyle modification
Dyslipidemia	HMG-CoA inhibitors (statins) Ezetimibe PCSK-9 inhibitors (eGFR >20 ml/min/1.73 m^2^) LDL cholesterol target values: – Patients with manifest atherosclerotic cardiovascular disease and very high-risk patients: LDL‑C <55 mg/dL and ≥50% reduction from baseline – High cardiovascular risk: LDL‑C <70 mg/dL and ≥50% reduction from baseline – Moderate/low cardiovascular risk: LDL‑C <116 mg/dL

*CKD* chronic kidney disease, *RAAS* renin–angiotensin–aldosterone-system, *LDL‑C* low-density lipoprotein cholesterol

### Smoking

Smoking has been linked to a higher risk of CKD progression and CKD-related CVD. As a result, patients with CKD who give up smoking see a reduction in albuminuria. All CKD patients should be urged to quit smoking and provided with the necessary smoking cessation programs [8].

### Dyslipidemia

The lipoproteins low-density lipoprotein (LDL) and high-density lipoprotein (HDL) are crucial in the progression of CVD in CKD patients. Due to this, KDIGO now advises all patients over the age of 50 with CKD stages 3–5 who are not on dialysis to get treatment with a statin or a statin/ezetimibe combination, regardless of their starting LDL-cholesterol (LDL-C) levels. In accordance with the European Society of Cardiology (ESC) recommendations, patients with CKD stage 3 should be treated toward an LDL‑C target of 70 mg/dL (ApoB 80 mg/dL; non-HDL‑C 100 mg/dL), while patients with CKD stage 4–5 should reach LDL‑C below 55 mg/dL (ApoB 65 mg/dL; non-HDL‑C 85 mg/dL; [9, 10]). The proatherogenic effects of LDL as well as of HDL in this patient population are particularly enhanced by different structural alterations (as discussed in more detail below). While there are several pharmacological alternatives for decreasing LDL‑C, including statins, ezetemibe, and PCSK9 inhibitors, the plasma level of HDL‑C and HDL function can currently not be medically addressed [9].

Lipid-lowering treatment is linked to a significantly lower risk of cardiovascular events and cardiovascular mortality in CKD patients who are not receiving dialysis. As demonstrated in a meta-analysis (*n* = 183.419), the risk of vascular events decreased by 21% (relative risk: 0.79; 95% confidence interval: 0.77–0.81) and the risk of (cardio)vascular mortality decreased by 12% (relative risk: 0.88; 95% confidence interval: 0.85–0.91) when a 1 mmol/L drug-induced drop in LDL‑C occurred. The meta-analysis also identified a significant interaction between the impact of statin medication and kidney function (*p* = 0.008; [11]). The benefit of beginning statin medication in CKD is diminished with worsening renal function and does not exist in individuals with CKD stage 5d.

Statin therapy in CKD patients is linked to an increased risk of myalgia (mostly limited to muscle pain), a rise in transaminases, or other side effects related to the muscle damage as occurs in the general population. The incidence of these side effects is higher [12], so that CKD patients frequently receive lower doses of statins associated with less LDL‑C lowering.

### Diabetes

Diabetes mellitus is a cause and a significant factor in the disease process of CKD. The Action to Control Cardiovascular Risk in Diabetes (ACCORD) study found that in the group of patients with impaired kidney function, strict blood sugar control (HbA1c 6.0%) compared to a standard blood sugar control (HbA1c 7.0–7.9%) was associated with a significantly higher all-cause (hazard ratio: 1.31; 95% confidence interval: 1.06–1.60) and cardiovascular mortality (hazard ratio 1.41; 95% confidence interval: 1.05–1.89). Strict blood glucose control is therefore not suitable for all our multimorbid patients. In order to prevent hypoglycemia, the 2022 KDIGO recommendations suggest an individual HbA1c target ranging from 7.0% to 8.0%. In younger patients with fewer comorbidities, the target of HbA1c is under 6.5% [13]. It should also be mentioned that many oral anti-diabetic medications for CKD patients must have their dosages altered to account for declining kidney function or they must be avoided in the case of advanced kidney disease. Blood glucose control in patients with diabetes type 2 and CKD should include lifestyle intervention, first-line therapy with metformin and an SGLT2 inhibitor and other medications if blood glucose control is inadequate [13].

### Hypertension

Arterial hypertension can be the result as well as the cause of CKD development and progression, thus creating a vicious circle. In the Systolic Blood Pressure Intervention Trial (SPRINT), the impact of different blood pressure targets—more aggressive (120 mm Hg systolic) versus more moderate (140 mm Hg systolic)—on cardiovascular events in non-diabetic adults was investigated. In the study, 28% of the patients had an eGFR of 20–60 mL/min/1.73 m^2^. By intensifying blood pressure control, there was a noticeable decrease in all-cause mortality (hazard ratio: 0.72; 95% confidence interval: 0.53–0.99) and cardiovascular events (hazard ratio: 0.72; 95% confidence interval: 0.53–0.99) in this subgroup [14]. By contrast, patients with diabetes who were randomly assigned to more stringent blood pressure control did not experience any appreciable benefits, according to the ACCORD study [15]. A blood creatinine concentration of less than 1.5 mmol/L was required for inclusion in this study [15].

Overall, it shows that the blood pressure target value in CKD patients should always be determined individually according to the known criteria and, if necessary, continuously adjusted in the course of the disease.

## Pathophysiological mechanisms contributing to increased cardiovascular risk in CKD

**Fig. 2:**
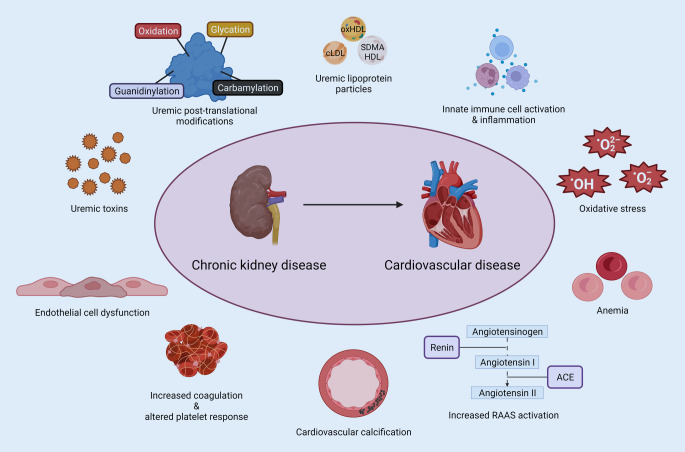
Chronic kidney disease (CKD)-specific pathophysiological and molecular mechanisms contributing to the accelerated cardiovascular disease (CVD) risk in CKD. *oxHDL* oxidized high-density lipoprotein, *cLDL* carbamylated low-density lipoprotein, *O*_*2*_^*2−*^ peroxide ion, *OH* hydroxide radical, O_2_^−^ superoxide anion, *ACE* angiotensin-converting enzyme. Created with Biorender

Beyond traditional cardiovascular risk factors, CKD-specific pathophysiological and molecular mechanisms have been identified that may contribute to increased risk of CVD in CKD. These include the accumulation of uremic toxins, CKD-associated post-translational modifications and uremic lipids, as well as chronic low-grade inflammation and oxidative stress, endothelial cell dysfunction, hypercoagulability and altered platelet responses, cardiovascular calcification, activation of the renin–angiotensin–aldosterone system (RAAS) and the sympathetic nervous system, and anemia (Fig. 2).

### Uremic toxins

Uremic toxins are metabolic products that increase in the circulation of CKD patients due to an increased production and/or an insufficient removal from the body due to a declined kidney function, up to such concentrations that they exert detrimental effects on organs and cells. To date, more than 140 uremic toxins have been identified. The EUTox database classifies these uremic toxins based on their physicochemical properties (small water-soluble, middle molecules, and protein-bound molecules) and lists the concentrations of uremic toxins as detected in patients with kidney failure (CKD stage 5d) compared to levels in healthy controls [16]. Uremic toxins were assigned a role in CKD progression as well as CVD development by contributing to chronic systemic inflammation, oxidative stress (by influencing molecular targets responsible for the generation of reactive oxygen species [ROS]) as well as endothelial cell dysfunction, thus negatively impacting vascular health [17–19].

### Uremic post-translational modifications and uremic lipoprotein particles

Post-translational modifications are alterations of proteins or peptides via the attachment of additional groups to the amino acid structure after protein translation and may impact protein function. Typical non-enzymatic post-translational modifications increased in CKD are carbamylation, guanidinylation, glycation, and oxidation, triggered by increased concentrations of, respectively, urea, guanidines, advanced glycation end products (AGEs), and ROS in CKD [20]. Each of these post-translational modifications has been associated with increased cardiovascular risk in CKD, with underlying mechanisms linked to processes of inflammation, oxidative stress, vascular damage, prothrombotic effects, and/or fibrosis.

For example, carbamylation of the intracellular sorting receptor sortilin in CKD increases vascular smooth muscle calcification and has been associated with enhanced cardiovascular calcification and increased CVD risk in CKD patients [21].

Apolipoprotein C3 (ApoC3) is guanidinylated in CKD patients. This increases its pro-inflammatory character and interferes with the regeneration capacity of damaged endothelium, which may underlie the association of ApoC3 guanidinylation with an increased progression of CKD as well as increased risk of cardiovascular events in CKD patients [22].

AGEs accumulate in CKD patients, even independent of hyperglycemia, triggered by a reduced clearance versus an increased production through increased oxidative stress. These AGEs have been shown to negatively impact vascular health, e.g., by triggering endothelial inflammation and oxidative stress, promoting endothelial dysfunction [19].

Oxidation as a result of increased oxidative stress can induce—among others—DNA damage, pro-inflammatory advanced oxidation protein products (AOPPs), as well as lipid oxidation products. Increased AOPP serum levels are associated with increased mortality risk in dialysis patients [23]. As an underlying mechanism, AOPP-modified albumin exerts pro-inflammatory effects on kidney, vascular, and cardiac cells [24–26], and may thus contribute to both CKD progression and CVD.

Also, lipoprotein particles are modified in composition in CKD via post-translational modifications as well as the association of uremic toxins or pro-inflammatory proteins, which can increase the pro-inflammatory character of lipoprotein particles (for LDL) or turn them from anti-inflammatory, protective lipoprotein particles to detrimental particles (for HDL; [27]). For example, LDL is increasingly oxidized and carbamylated with CKD progression, which in turn triggers ROS production and endothelial cell activation, favoring the development of CVD [27]. Also, HDL is oxidized and carbamylated in CKD, and it accumulates the uremic toxin SDMA and the acute phase protein SAA, all conferring a pro-inflammatory character to HDL [27].

### Innate immune cell activation, inflammation, and oxidative stress

Patients with CKD display a chronic low-grade inflammation initiated by unresolved kidney damage, which triggers innate immune activation and systemic inflammation due to an increasing uremic milieu in the body. In patients with CKD, increased systemic levels of the pro-inflammatory proteins high-sensitivity C‑reactive protein (CRP), interleukin (IL)-6, and tumor necrosis factor (TNF)-α, as well as of monocyte surface levels of IL-1α correlated with increased cardiovascular risk, underlining inflammation as an important cardiovascular risk factor in CKD [28]. Monocytes and endothelial cells are key contributors to the increasing levels of inflammatory cytokines in the blood [29]. Monocytes of CKD patients express high levels of interleukin (IL)-1β and IL-1α, with IL-1β associated with an increased risk of atherosclerosis [30]. IL-1β is produced by activation of the inflammasome, and the CANTOS (Canakinumab Anti-inflammatory Thrombotic Outcomes Study) trial revealed that inhibiting IL-1β is promising in ameliorating cardiovascular outcome in both the general population as well as CKD patients [31]. Also, IL-1α was found to be an important mediator of increased inflammatory risk in CKD by triggering endothelial inflammation and immune cell adhesion, and by promoting tissue infiltration of pro-inflammatory neutrophils and monocytes/macrophages in inflammatory conditions [30]. Endothelial cells further contribute to an inflammatory state in CKD upon endothelial activation by the uremic milieu [19], triggering the secretion of, for example, IL‑1, IL‑6, and monocyte chemoattractant protein‑1 [32], all potentiating the inflammatory response and favoring the development of CVD.

Oxidative stress in CKD develops through the increased production of ROS such as hydrogen peroxide and/or superoxide due to processes of mitochondrial dysfunction, endoplasmic reticulum stress, and activation of the NOX enzyme, leading to an imbalance of pro-oxidative vs. antioxidative agents. Oxidative stress contributes to the progression of kidney damage (by impacting inflammation and fibrotic changes) as well as to increased cardiovascular risk in CKD (through processes of endothelial cell dysfunction and immune cell activation, among others) [33].

### Endothelial dysfunction

Endothelial cells are the gatekeepers between the blood and the vasculature, and thus important regulators of vascular health. Factors such as inflammation, oxidative stress, uremic toxins, and hypertension as present in CKD can induce endothelial cell inflammation and dysfunction, resulting in an increased cardiovascular risk [4]. Characteristics of endothelial dysfunction include enhanced vascular permeability, vascular inflammation, increased leukocyte–endothelium interactions, pro-thrombotic properties, and impaired vasodilation [34]. Under CKD conditions, endothelial cells display a shift toward a pro-inflammatory, pro-atherosclerotic, pro-thrombotic, and pro-vasoconstrictive phenotype, with involved molecular pathways discussed in detail in the review by Baaten et al. [4].

### Increased coagulation and altered platelet responses

By and large, CKD conditions affect the coagulation system by triggering an upregulation of procoagulant factors such as tissue factor and a downregulation of anticoagulant proteins such as protein C and S. Overall, CKD patients display hypercoagulability, which is associated with an increased risk of thrombosis and thus higher cardiovascular mortality [35].

Both thrombotic and hemorrhagic complications occur in CKD patients, suggesting also abnormalities in the platelet response. A systematic review and meta-analysis by Baaten et al. on platelet function in CKD revealed opposing findings: On the one hand, platelet adhesion was mostly found to be reduced and bleeding time prolonged, reflecting impaired platelet function. On the other hand, some studies showed increased platelet aggregation responses or found platelet function to be unchanged in CKD. These conflicting results might be due to heterogeneity in the disease status, which remains to be clarified in further studies [36].

### Cardiovascular calcification

Cardiovascular calcification leads to advanced vascular aging and cardiovascular risk. Patients with CKD display a disturbed mineral homeostasis (summarized as CKD-mineral and bone disorder) including an abnormal mineral metabolism and abnormal bone and extraskeletal calcification. Calcification of the medial layer of central and peripheral arteries is typically found in CKD patients—whereas it is mainly absent in people with healthy kidney function < 60 years of age—and is associated with increased arterial stiffness and cardiovascular mortality [37]. Medial calcification involves processes of mineral nucleation and growth as well as osteogenic differentiation of vascular smooth muscle cells. The molecular pathways involved in cardiovascular calcification are complex and neither early biomarkers nor therapies consistently reducing cardiovascular calcification and cardiovascular outcome in CKD are clinically available to date [37].

### Renin–angiotensin–aldosterone and sympathetic nervous system activation

The RAAS is essential for regulating blood volume, electrolyte and fluid balance, and vascular resistance [38]. Chronic kidney disease is associated with a chronic activation of the RAAS as well as of the sympathetic nervous system (SNS), which results in persistently increased blood flow and hypertension. Inhibition of the RAAS system under CKD conditions is cardioprotective as shown in animal models of uremic cardiomyopathy, in which RAAS inhibition prevented or at least alleviated CKD-induced cardiac fibrosis and cardiac inflammation, and this at least partly also through blood pressure-independent effects [7].

Activation of the RAAS system is associated with activation of sympathetic nerve activity, which leads to the release of neurotransmitters such as norepinephrine or catecholamines. These neurotransmitters can contribute to hypertension, vascular stiffness, and endothelial dysfunction [39]. Inhibition of increased SNS activity in a cardiorenal rat model reduced blood pressure, cardiac fibrosis, and cardiac apoptotic pathways [7].

### Anemia

Multiple factors contribute to the development of anemia in CKD, such as the reduction of endogenous erythropoietin within the kidneys, in addition to factors such as iron deficiency, impaired iron absorption, systemic inflammation as well as uremic toxins [40].

The observational study NADIR‑3 revealed that CKD3 patients who developed anemia had a faster progression of CKD, higher rates of hospitalization, as well as an increased cardiovascular risk compared to patients without anemia [41].

## Novel therapeutic strategies and perspectives

As in the general population, traditional cardiovascular risk factors remain of strong clinical relevance in the CKD population and form the basis of current therapeutic treatment to reduce cardiovascular risk. However, increased cardiovascular risk in patients with CKD along with alterations in CVD presentation and outcome is triggered also by CKD-associated pathophysiological mechanisms that on the molecular level are—at least in part—different from those in non-CKD patients. Triggered by a reduced kidney function and an increased uremic environment with uremic toxins and uremic lipids—among others—CKD patients show a chronic low-grade inflammation and an increased innate immune activation, an increased activation of the RAAS and the sympathetic nervous system, along with increased endothelial dysfunction, medial calcification, vascular stiffness, as well as hypercoagulability.

Recent clinical trials have shown a benefit of reducing chronic low-grade inflammation to reduce cardiovascular risk, also in CKD patients. In the CANTOS trial, canakinumab (an IL-1β targeting antibody) reduced the number of major cardiovascular events in CKD patients with a prior myocardial infarction and high inflammatory (CRP) status [31]. In the RESCUE trial, ziltivekimab (an IL‑6 targeting antibody) reduced biomarkers of inflammation and thrombosis in CKD patients with high CRP levels [42]. Furthermore, both sodium-glucose cotransporter‑2 (SLGT2) inhibitors and glucagon-like peptide 1 receptor agonists (GLP1RA) as established anti-diabetic therapies exert anti-inflammatory effects and revealed kidney as well as cardiovascular protection, also independent of glucose control [28, 43]. Finerenone—a non-steroidal, selective mineralocorticoid receptor antagonist that reduced CKD progression and cardiovascular events in patients with diabetic CKD—was also linked to anti-inflammatory potential [28, 43]. Thus, anti-inflammatory strategies reveal promise in reducing cardiovascular risk also in CKD patients.

## Conclusion

Unraveling in detail the disease mechanisms involved in chronic kidney disease (CKD) is important for further development and optimization of therapeutic strategies to reduce cardiovascular risk, tailored to the CKD population. Also, this could support improved patient stratification by identifying patient subcohorts that are particularly prone to benefit from specific therapies, such as, e.g., targeted anti-inflammatory therapies. Future preclinical and clinical studies are expected to provide deeper insights into the potential of novel anti-inflammatory therapies to reduce increased cardiovascular risk in CKD.

## Declarations

For this article no studies with human participants or animals were performed by any of the authors. All studies mentioned were in accordance with the ethical standards indicated in each case.
